# A novel prognostic scoring model based on cuproptosis identifies COMMD1 as a novel therapy target for liver hepatocellular carcinoma

**DOI:** 10.32604/or.2024.049772

**Published:** 2025-02-28

**Authors:** KE TIAN, ZHIPENG LI, XIANGYU ZHAI, HUAXIN ZHOU, HUI YAO

**Affiliations:** 1 General Surgery Department 2, The No. 2 People’s Hospital of Lanzhou, Lanzhou, 730030, China; 2 The Hepatobiliary Surgery Department, The Second Hospital of Shandong University, Jinan, 250000, China; 3 Organ Transplant Department, Qilu Hospital of Shandong University, Jinan, 250000, China

**Keywords:** Cuproptosis, Hepatocellular carcinoma (HCC), Copper homeostasis, Prognostic model, Immunocytes

## Abstract

**Background:**

Primary liver cancer poses a significant global health burden, with projections indicating a surpassing of one million cases by 2025. Cuproptosis, a copper-dependent mechanism of cell death, plays a crucial role in the pathogenesis, progression, and prognosis of various cancers, including hepatocellular carcinoma (HCC).

**Purpose:**

This study aimed to develop a prognostic model for HCC based on cuproptosis-related genes, utilizing clinical data and gene expression profiles from The Cancer Genome Atlas (TCGA) and Gene Expression Omnibus (GEO) databases.

**Materials and Methods:**

Clinical features and gene expression data of HCC patients were collected from publicly available databases. Patients from TCGA were randomly divided into training and testing sets, and Lasso Cox regression was applied to develop a predictive model using cuproptosis-related genes.

**Results:**

The analysis identified Copper Metabolism Domain Containing 1 (COMMD1) as a potential prognostic marker for HCC, with deletion of this gene impacting disease progression. Cellular functional experiments validated the role of COMMD1 in HCC.

**Conclusions:**

COMMD1 emerges as a promising candidate for HCC treatment, with implications for prognosis prediction and therapeutic targeting.

## Introduction

HCC represents 75%–85% of primary liver cancer cases and is the fifth most common cancer globally, ranking third in cancer-related mortality. Annually, approximately 5 million new cases are reported [1]. In addition to HCC, primary liver cancer also consists of intrahepatic cholangiocarcinoma (10%–15%) and other less common forms. Thanks to advancements in vaccines and treatments, the incidence of liver cancer caused by Hepatitis B virus (HBV) and Hepatitis C virus (HCV) is decreasing [2]. There has been a rise in liver cancer linked to alcohol, tobacco, and prolonged aflatoxin exposure [3,4]. Limited research exists on breast cancer metastasizing to the liver within the scientific community. Patients experiencing liver metastases face a grim prognosis, enduring a median survival period of merely 14–16 months irrespective of treatment modalities [5]. The effectiveness of ICIs as primary or secondary treatments for HCC did not result in a notable enhancement in patient outcomes [6,7]. Nevertheless, given the diversity of HCC, the effectiveness of current primary or secondary medication remains inadequate [6]. Hence, identifying new therapeutic targets linked with HCC is imperative to enhance treatment outcomes and devise innovative therapeutic strategies.

Cuproptosis, an emerging form of cell demise, emerges due to disruptions in copper ion metabolism, triggering Dihydrolipoyl Transacetylase **(**DLAT) oligomerization. This leads to the depletion of iron-sulfur cluster proteins, causing cell death from proteotoxic stress [8]. Copper is a vital micronutrient for all life forms, prone to redox reactions, and serves as electron transfer within the oxidative respiratory chain of the Tricarboxylic Acid Cycle (TCA) cycle. Mutations in the ATP7B gene can cause copper accumulation in the liver and brain’s nucleus accumbens, leading to liver failure, ataxia, and other issues [9]. Elesclomol, acting as a carrier of copper ions, can transport them into cells and has been shown to induce cuproptosis [10]. In the Phase III clinical trial, two treatment regimens, comprising elesclomol combined with paclitaxel and paclitaxel alone, were evaluated in chemotherapy-naive patients. The results demonstrated a significantly elevated PFS in the combination testing group [9]. Elesclomol, known for inducing cuproptosis, has demonstrated efficacy in eliminating cisplatin-resistant lung cancer cells [11]. Additionally, it causes cuproptosis in colorectal cancer cells by knocking down ATP7A. The correlation between mitochondrial metabolism and their susceptibility to elesclomol was observed [12,13]. Also, related studies have documented a notable rise in mitochondrial metabolism in hepatocellular carcinoma cells [14]. A recent study about cuproptosis showed that HCC can be significantly inhibited by elesclomol [15]. These studies suggest that hepatocellular carcinoma may be sensitive to cuproptosis.

In this study, we performed silico analyses and basic biological experiments, and suggested that alterations in the expression of COMMD1 play a key role in tumorigenesis, progression, and prognosis, and COMMD1 can be used as a predictor of tumor prognosis.

## Materials and Methods

### Data acquisition

Transcriptome data and prognostic information were retrieved from The Cancer Genome Atlas (TCGA) database (TCGA-HCC: https://portal.gdc.cancer.gov/repository, accessed on 15/03/2024) and the GEO database (GSE76427) (http://www.ncbi.nlm.nih.gov/geo, accessed on 15/03/2024).

### Identification of cuproptosis-related genes

We employed the STRING website (https://string-db.org/) using the keywords “cuproptosis” and “copper metabolism” to obtain the expression relationships of relevant genes (The 14 genes of “cuproptosis” and “copper metabolism” are listed in Fig. S1). Moreover, a PubMed search was conducted to identify genes meeting the study criteria.

### Cuproptosis-related gene prognostic model construction and verification

To mitigate overfitting, Lasso Cox regression was first employed in the prognostic risk model. The risk score was then computed using coefficients from multivariate Cox regression analysis. Kaplan–Meier analysis validated its efficacy as an independent prognostic predictor. ROC curve analysis assessed the model’s predictive accuracy.

### Clinical information analysis

Cox analyses, both univariate and multivariate, were conducted using the R programming language (version 4.2.1). Survival data for patients was displayed using the forest plot tool along with calibration curves for survival rates at 1-, 3-, and 5-year.

### Molecular subtype grouping analysis

Initially, correlation analysis was performed on 14 copper metabolism-related genes, visualized via correlation chord diagrams. Subsequently, cluster analysis was conducted with k = 2 using the Consensus matrix. Additionally, ssGSEA analysis was performed on the six selected cuproptosis-related genes for molecular subtype grouping using the ‘GSVA’ R package.

### Immuno-infiltration analysis

Immune infiltration in high- and low-risk patient groups was assessed using CIBERSORT analysis in R, specifically with the CIBERSORT R script (v1.03) [16]. The Spearman correlation method analyzed risk score correlations within each group.

### GO analysis and KEGG analysis

GO and KEGG analyses enriched biological functions and pathways for gene expression in these two groups. Visualization utilized the ggplot2 package.

### Drug sensitivity analysis

Data on cell lines, gene expression, and drug sensitivity were obtained from the Genomics of Drug Sensitivity in Cancer (www.cancerrxgene.org, accessed on 15/03/2024). The analysis utilized the “oncoPredict” package in R, with visualization conducted using the “ggplot2” package [17].

### Cell culture and transfection

Hep3B and Huh7 cells were cultured in DMEM complete medium at 37°C with 5% CO_2_. Four COMMD1-siRNAs and corresponding negative control (NC) siRNAs were obtained from General Biol to silence COMMD1 expression. Cells were seeded in 6-well dishes (1 × 10^5^ cells per well) until reaching 30%–50% confluence before transient transfection. Liposome 2000 was used for transfection at a concentration of 100 nM. Gene expression analysis was conducted 48 h post-transfection. Tetrathiomolybdate (TTM) was also utilized in the study.

Human HCC cell lines were sourced from the Cell Bank of the Chinese Academy of Sciences (Shanghai, China). Each cell line underwent short tandem repeat (STR) analysis for verification and was tested for mycoplasma contamination prior to experiments.

### RNA extraction and qPCR

Total RNA was extracted using Fastagen kits (9109, Takara, Japan) as per the manufacturer’s instructions. cDNA synthesis involved incubation at 37°C for 15 min, followed by denaturation at 85°C for 5 s and cooling at 4°C for 5 min, using the PrimeScriptTM RT kit (RR036A, Takara, Japan) with gDNA Eraser. qPCR analysis was used by the SYBR Premium Delivery TaqII® system according to the manufacturer’s instructions. Gene expression levels were determined using the 2^−ΔΔCt^ method relative to a control group. Sequences of primers and internal references are as follows: Homo-COMMD1-149F TCAGTCAAGGCACTCAGCTC Homo-COMMD1-149R CTTCTACCTCTGACAGCGTCT Homo-ACTB-176F GAGAAAATCTGGCACCACACC Homo-ACTB-176R GATAGCACAGCCTGGATAGCAA

### Cell proliferation and Transwell assays

Cell proliferation was assessed using CCK-8 (C0038, Beyotime, China) and EdU (C0071S, Beyotime, China) assays, following the manufacturer's instructions. For the EdU assay, dilute the EdU working solution to 2× (20 μM) with cell culture medium, then add it to the culture plate being tested (1:1 with the medium in the plate), resulting in a final EdU concentration of 1× (10 μM) in the wells. The Transwell assay assessed HCC cell invasion and migration. Cells were added to the upper chamber, with serum-free medium, and the lower chamber contained medium with 10% fetal bovine serum. After fixation and staining, images were captured, and cell penetration was quantified.

### Copper assay

The copper assay kit (E-BC-K300-M, Elabscience) from Wuhan, China was used to measure copper levels in cell lysates following the manufacturer’s instructions. Absorption was then measured using the Cytation 5 Cell Imaging Multi-Mode Reader at a wavelength of 575-585 nm from Agilent in the United States.

### Western blot assay

Cells were lysed in RIPA buffer with 1× protease inhibitor and homogenized on ice for 30 min. After centrifugation at 12,000 RPM for 15 min to remove debris, total protein was quantified using a BCA Protein Assay Kit (P0012, Beyotime, China). Samples were mixed with 5× loading buffer, boiled for 10 min, and separated on a 10% SDS-PAGE gel (80 V for 30 min then 120 V for 55 min). Proteins were transferred to PVDF membranes (300 mA for 105 min), blocked with 5% skimmed milk, and incubated with primary antibodies overnight at 4°C. After washing with TBST, membranes were incubated with secondary antibodies, washed again, and developed using ECL solution. Imaging was performed with a Tanon 4800 machine. Antibodies used: COMMD1 (Proteintech 11938-1-AP), DLAT (Abcam ab172617), Beta Actin (Proteintech 81115-1-RR).

### Statistical analysis

Statistical analysis was performed using SPSS 17.0 (IBM, Chicago, IL, USA) and GraphPad Prism 8.0 (GraphPad Software, San Diego, CA, USA). Data are presented as means ± standard deviations (SD). Group comparisons were made using both the *t*-test and one-way ANOVA. Additionally, comparisons among the three groups were assessed using ANOVA and *t*-tests with LSD for *post hoc* analysis. Significance levels were denoted as follows: *p < 0.05, **p < 0.01, ***p < 0.001, and ****p < 0.0001, n.s. for not significant.

## Results

**Figure 1:**
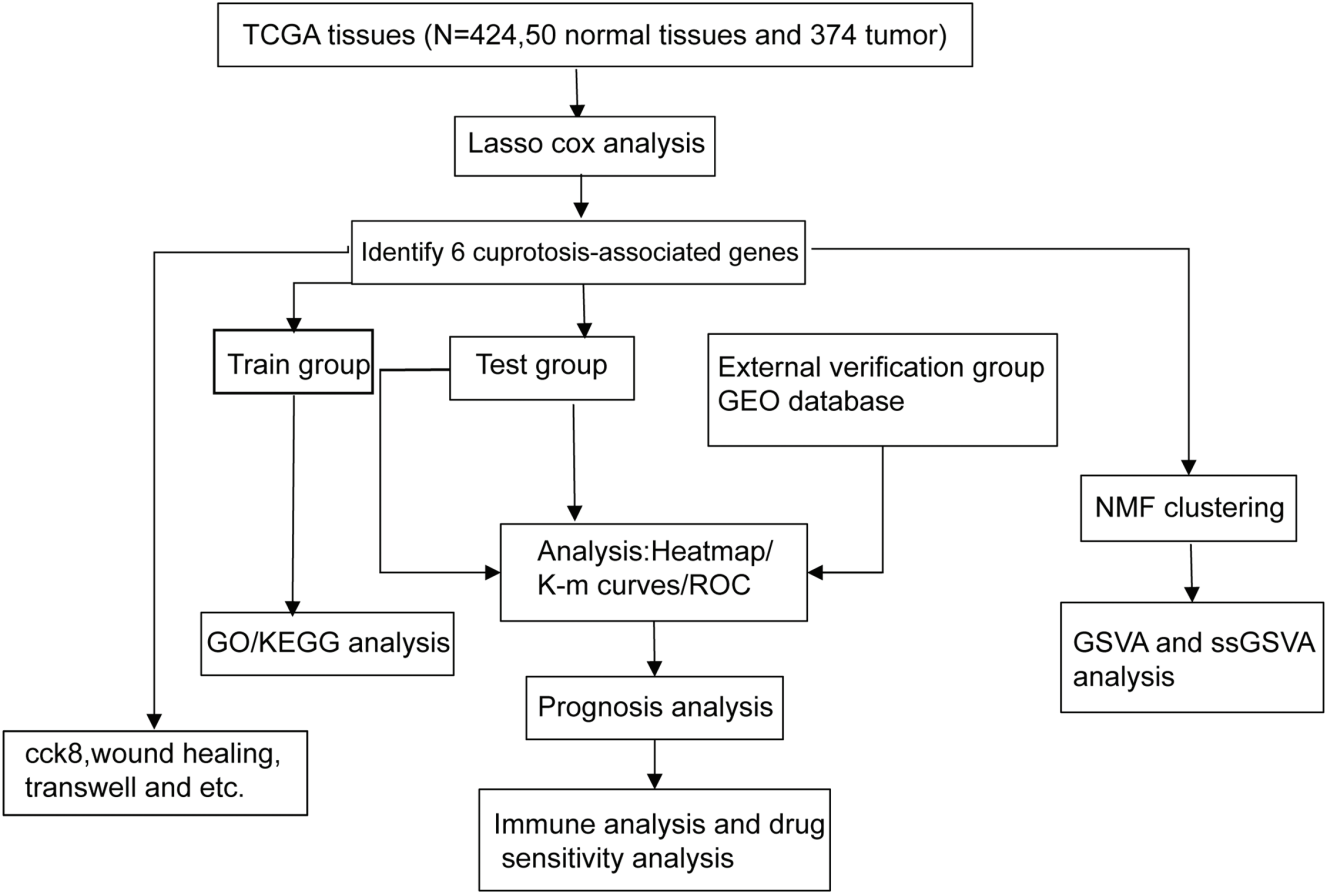
The flow chart shows the overall experimental design of this study.

The following figure delineates the comprehensive experimental design utilized in this investigation (Fig. 1).

### Prognostic model construction for cuprotosis-associated genes using the TGCA dataset of the HCC

The prognostic model for hepatocellular carcinoma was established using data from the TCGA database. Initially, patients of the TCGA were divided into two groups randomly: a training group comprising 118 cases and a testing group comprising 115 cases. Subsequently, lasso Cox regression analysis (Fig. S2) was conducted to identify cuproptosis-associated genes and formulate risk scores based on these genes. Risk score = “FDX1”*“−0.268195356896634” + “CAT”*“−0.00532048817334454” + “CDKN2A”*“0.14379580824953” + “DLAT”*“0.4566822764627” + “LIPT1”*“0.512540028122221” + “COMMD1”* “0.26820835910616”.

**Figure 2:**
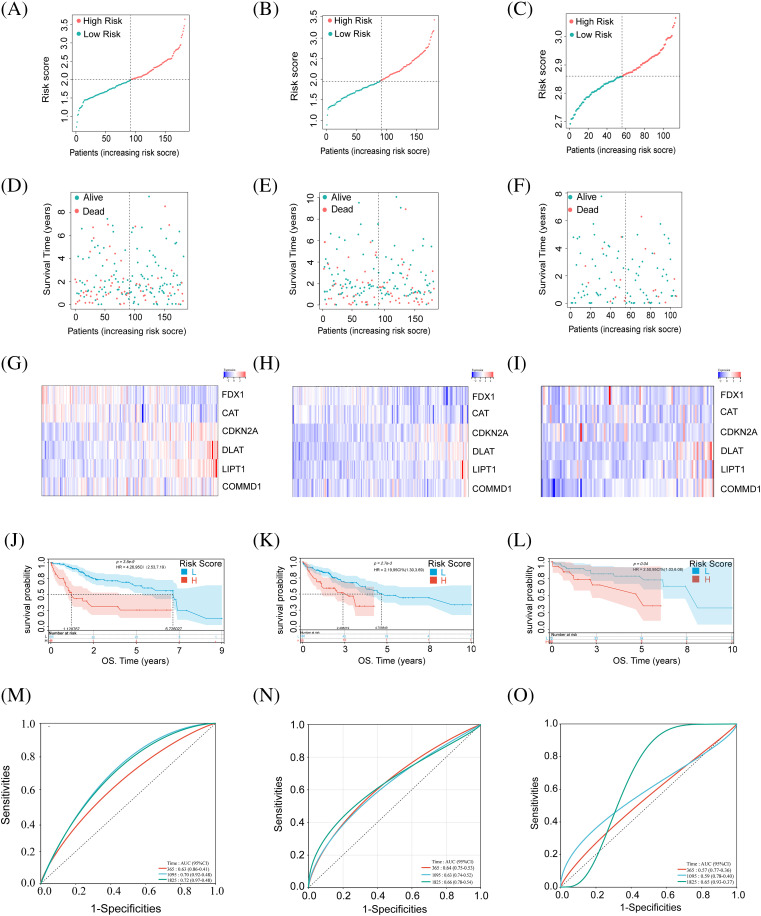
Identification of CRGs in relation to the prognosis of HCC patients in the training set, test set, and external validation set. (A–C) Risk score of the training, testing, and external validation set. (D–F) Association between risk score and survival status in the training, testing, and external validation set. (G–I) The heat map of six CRG expressions in training, testing, and external validation set. (J–L) Kaplan–Meier curves of survival in training, testing, and external validation set. (M–O) ROC curves for the prognostic prediction of risk score model at 1-, 3- and 5-year survival times in training, testing, and external validation set.

Patients were categorized into high and low-risk groups based on calculated scores (Fig. 2A,B). Low-risk HCC patients had better outcomes than high-risk ones in both training and validation cohorts (Fig. 2D,E). Heatmaps showed gene expression in these two groups (Fig. 2G,H). Survival curves in both cohorts had significant *p*-values (Fig. 2J,K). There was significant stratification between these two groups in both cohorts. ROC curves (Fig. 2M,N) indicate model efficacy in predicting HCC prognosis.

### Additional validation of the prognostic model

Using the GEO dataset (GSE76427), we applied our risk score formula, stratifying the external validation set into high- and low-risk groups based on median risk scores from the training set (Fig. 2C). Survival status and gene expression heatmaps for the six cuproptosis-related genes were analyzed (Fig. 2F,I). According to previous research, the high-risk group patients had a worse prognosis than the other group (Fig. 2L). ROC analysis for OS at 1, 3, and 5 years was performed (Fig. 2O). External validation confirmed the reliability of our prognostic model.

### Assessing other prognostic factors

**Figure 3:**
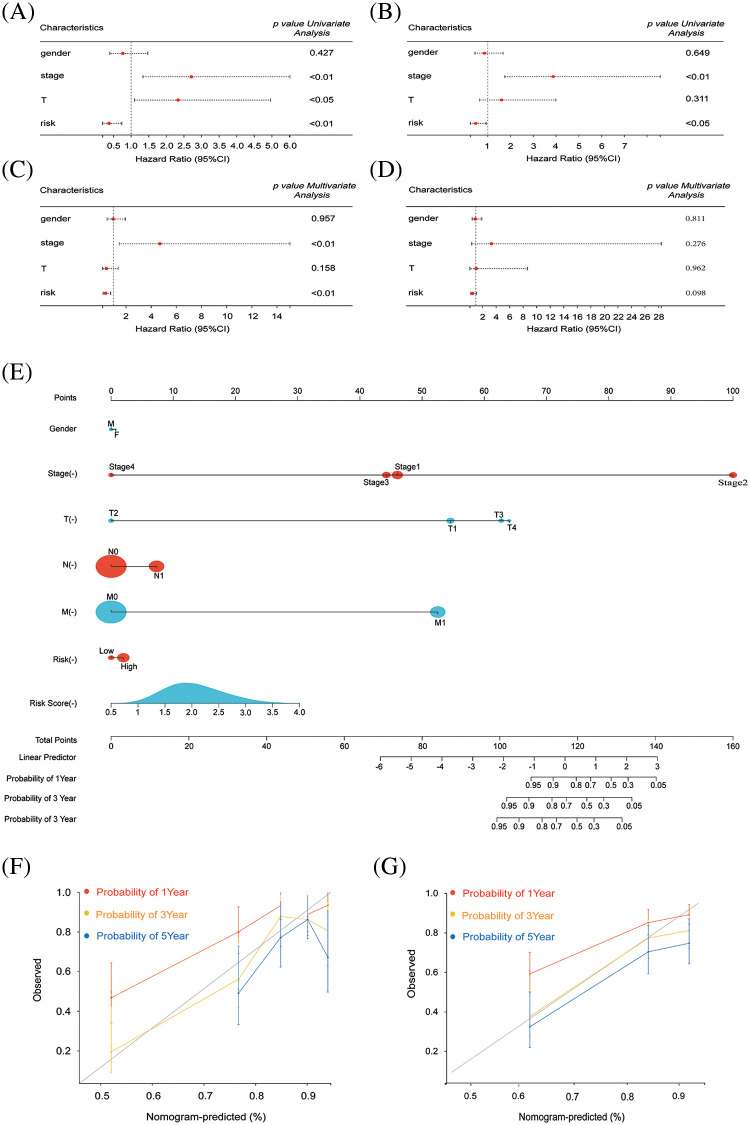
Survival and prognosis analysis of HCC patients in TCGA. (A and B) Univariate Cox regression analysis was performed to evaluate the association between risk scores, clinical parameters, and overall survival (OS) in both the training and test groups of patients with HCC. (C and D) Multivariate Cox regression analysis was conducted to assess the impact of risk scores, clinical parameters, and overall survival (OS) in patients with HCC in both the training and test groups. (E) A combined nomogram for the risk score model and 1-, 3-, and 5-year survival rate. (F) The calibration plot of the modeling group for predicting patient survival at 1, 3, and 5 years. (G) The calibration plot of the testing group for predicting patient survival at 1, 3, and 5 years.

Following validation of our prognostic model, the assessment extended to include other independent prognostic factors, such as gender, tumor stage, and size, which may exert influence on patient prognosis. In the training group, one-way Cox analysis confirmed the potential utility of tumor stage and tumor size as independent prognostic factors. Our risk score was validated as an independent prognostic factor (Fig. 3A). Multifactor Cox analysis in the training group identified tumor stage as another potential independent prognostic factor (Fig. 3B). Univariate and multivariate Cox analyses were performed in the test group, confirming tumor stage as a significant independent prognostic factor (Fig. 3C,D). Nomogram curves were generated to depict the relationship between patient gender, tumor stage, 1-, 3-, and 5-year survival, and other risk factors with risk scores (Fig. 3E). Survival curves for 1-, 3-, and 5-year periods in both testing and modeling groups demonstrated accurate prediction of patient survival (Fig. 3F,G).

### Focusing cuproptosis genes in HCC

**Figure 4:**
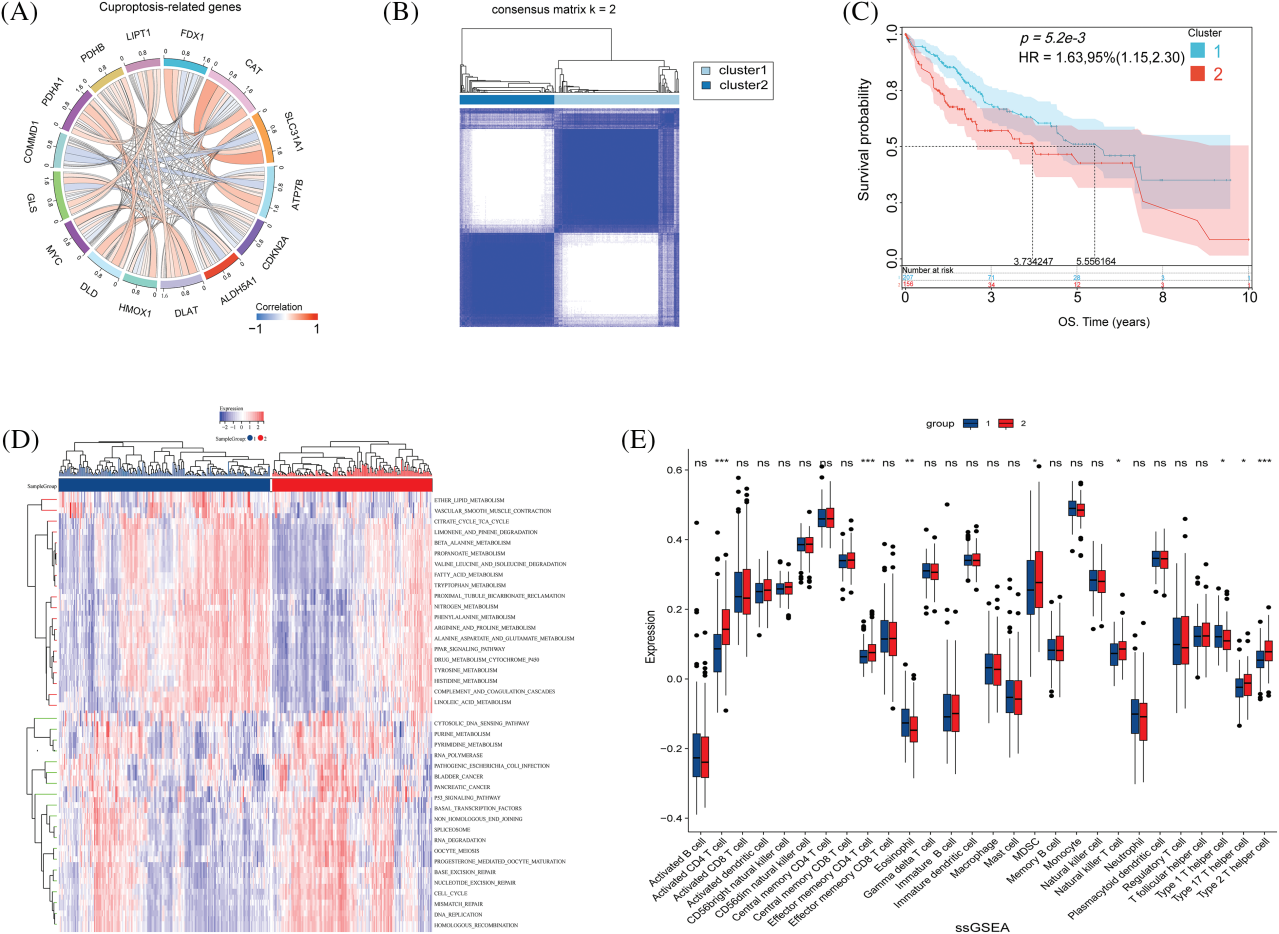
Biological characteristics of cuproptosis subtypes in HCC. (A) The relationship in cuproptosis-associated genes in HCC (The connecting lines represent their interactions, and the color of the lines represents the strength of the correlation). (B) Consensus matrix heatmap defining two clusters (k = 2). (C) Kaplan–Meier curves of survival in the two clusters. (D) Heatmap of GSVA analysis between the two clusters. (E) The boxplot shows the proportion of the 22 types of immune cells in HCC patients from the two clusters. The *p*-values were set as *p < 0.05, **p < 0.01, ***p < 0.001, n.s. for not significant.

Initially, FDX1, LIPT1, COMMD1, DLAT, CAT, and CDKN2A were identified as pivotal cuproptosis genes, leveraging insights from existing studies. Following this, the TCGA database illustrated the relationships among cuproptosis-related genes (Fig. 4A). Patients were classified into two subtypes based on cuproptosis-related gene expression using NMF (Fig. 4B). Survival curves for the two subtypes showed a significant difference (*p* < 0.001) (Fig. 4C). GSVA analysis found that subtype 1 had significantly increased expression in pathways such as lipid metabolism and the tricarboxylic acid cycle. In contrast, subtype 2 had increased expression in the p53 pathway and pancreatic cancer (Fig. 4D). Finally, ssGSEA analysis was conducted on the two subtypes to evaluate disparities in immune expression. Subsequently, significant differences were observed in CD4^+^ T cell and TH2 cell expression between the subtypes, with notably higher expression levels of these two kinds of observed in subtype 2 compared to subtype 1 (Fig. 4E). CD4^+^ T cells play their unique role in tumor progression. Their role in cuproptosis remains to be further confirmed.

### Immunological profile of HCC patients

**Figure 5:**
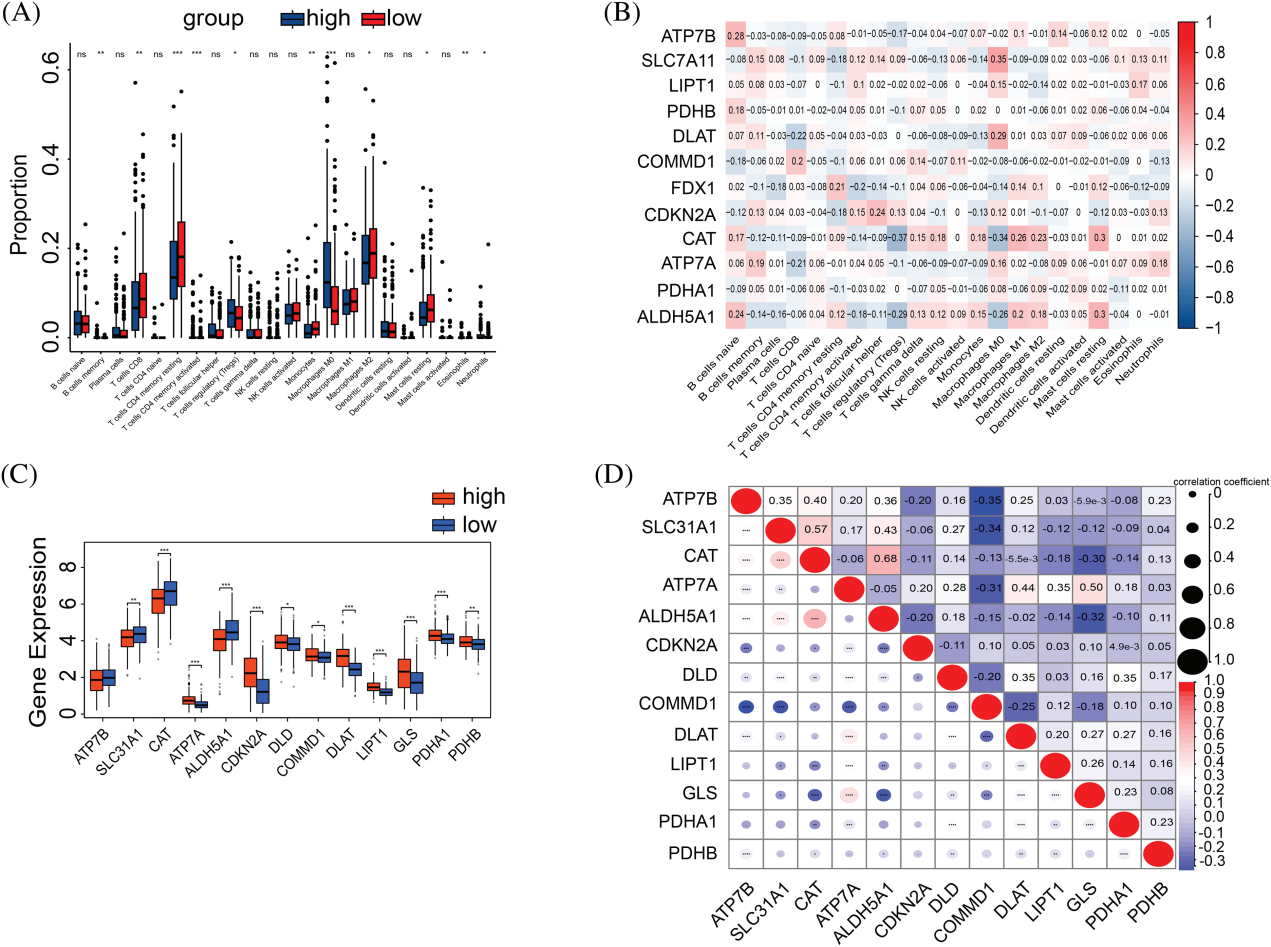
Correlation between tumor immune and risk. (A) The boxplot shows the results of the CIBERSORT analysis between high and low-risk groups. (B) The correlation graph of cuproptosis-related gene expression in relation to immune cells. (C) The expression in immune checkpoint genes between high-risk and low-risk groups. (D) Spearman correlation between immune checkpoints and risk scores. The *p*-values were set as *p < 0.05, **p < 0.01, ***p < 0.001, and ****p < 0.0001, n.s. for not significant.

Tumor immunity is pivotal in tumor progression, and its dysregulation, often due to immune escape mechanisms, can precipitate metastasis. CIBERSORT analysis compared immunization groups with different cuproptosis-related gene expression (Fig. 5A). Significant differences were found in CD8^+^/CD4^+^ T cells and Treg cells. The high-risk group showed elevated Treg cell expression and lower levels of CD8^+^/CD4^+^ T cells, critical for tumor immunity. Moreover, Treg cells serve as pivotal immunomodulatory entities in tumor immunity, representing a significant component in immune evasion within tumor tissues. Elevated Treg cell expression in high-risk patients implies a heightened potential for evading tumor immune responses. Then, we explored the correlation between cuproptosis genes and immune cells (Fig. 5B).

The high-risk cohorts showed reduced expression of FDX1, SLC31A1, CAT, ATP7B, and ALDH5A1, and elevated expressions of CDKN2A, DLAT, LIPT1, PDHA1, and COMMD1 compared to the low-risk group (Fig. 5C). Moreover, COMMD1 displayed a negative correlation with the expressions of ATP7B, SLC31A1, and DLAT (Fig. 5D).

### Enrichment analysis of gene pathways

**Figure 6:**
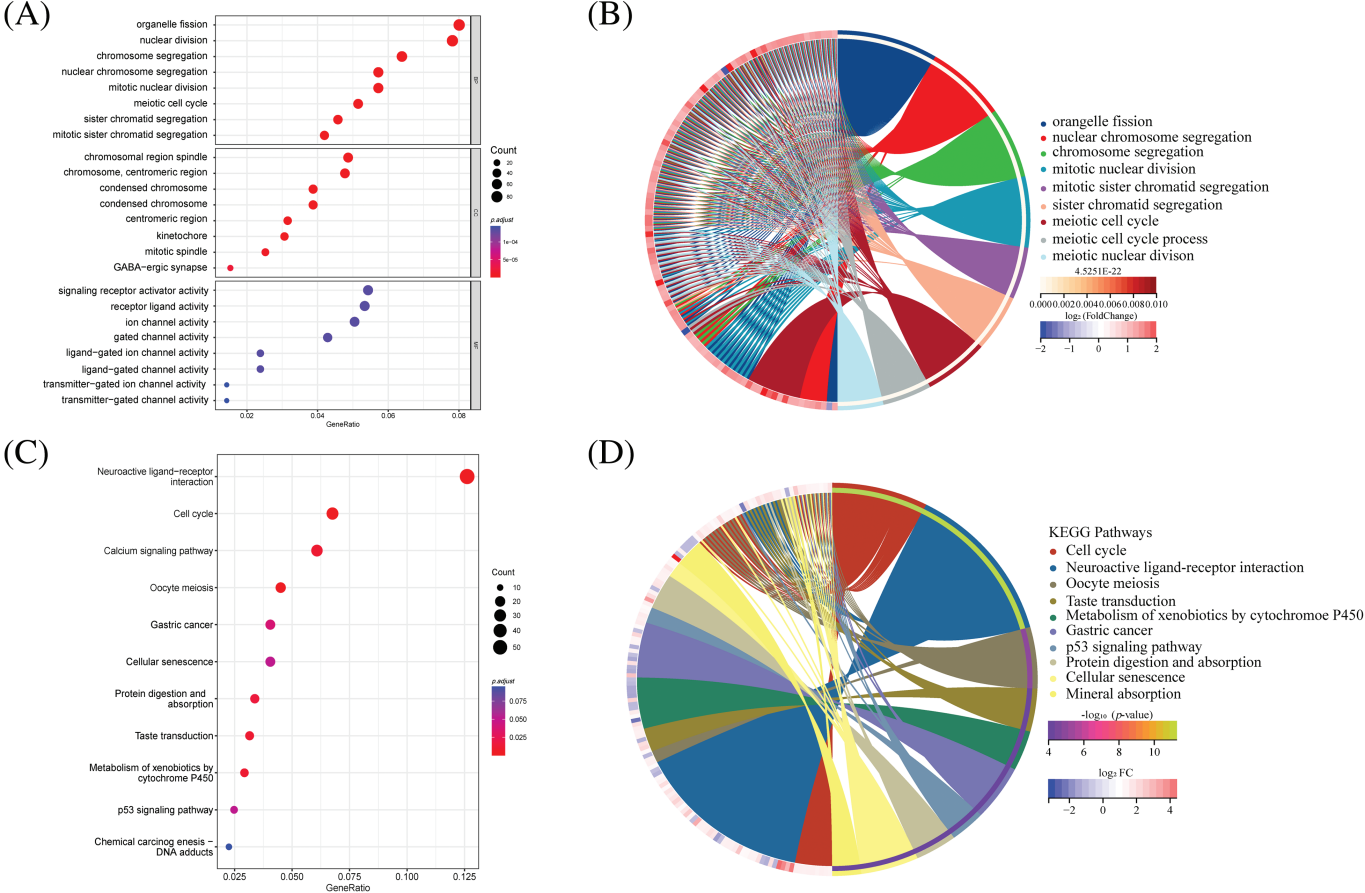
GO and KEGG analysis in differential genes between high-risk and low-risk groups. (A) The bar plot displaying GO enrichment analysis reveals the top three significantly enriched terms in cell cycle-related categories. (B) The circos plot of interconnection between GO terms. (C) The bar plot of KEGG enrichment analysis. (D) The circos plot of interconnection between KEGG terms.

GO and KEGG analyses were performed. The GO analysis unveiled the enrichment of DNA replication and cell division pathways among the differential gene pathways observed in both high-risk and low-risk groups (Fig. 6A). Additionally, the circus plot illustrates the relationship between the enriched pathways and their associated genes in the GO analysis (Fig. 6B). Conversely, KEGG analysis highlighted significant enrichment of receptor interaction pathways, cell cycle, and calcium signaling pathways (Fig. 6C). The circus plot (Fig. 6D) demonstrates the relationship between enriched pathways and related genes.

### Drug sensitivity of patients in high- and low-risk groups

To assess the sensitivity of patients in the high/low-risk groups to various chemotherapeutic agents, we obtained relevant datasets from the GDSC. Subsequently, we identified and visualized 16 sensitive drugs (Fig. S3). The results indicated that drugs such as camptothecin, IGF1R inhibitors, Mirin, and Irinotecan exhibited greater efficacy in the high-risk group.

### Identification of COMMD1 as a gene that can be used as a predictor of HCC prognosis

**Figure 7:**
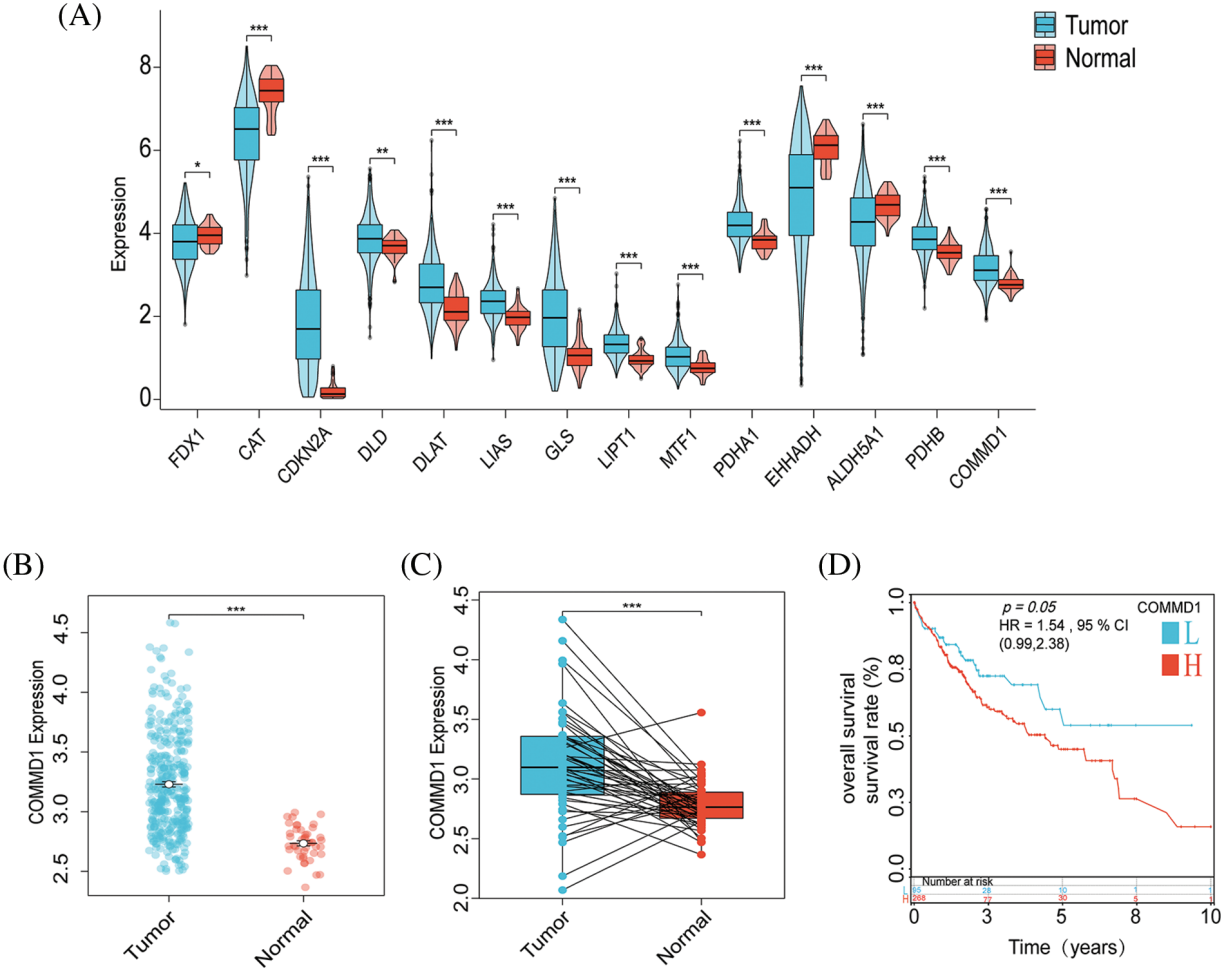
COMMD1 is a prognosis marker for HCC. (A) Gene expressions for CRGs. (B) The expression of COMMD1 in TCGA. (C) Relative expression of COMMD1 in TCGA. (D) OS curve between high- and low-expression of COMMD1. The *p*-values were set as *p < 0.05, **p < 0.01, ***p < 0.001, n.s. for not significant.

Our initial step involved a thorough differential analysis of CRGs, aiming to pinpoint genes with potential prognostic genes for HCC (Fig. 7A). This showed that COMMD1, In tumors, CDKN2A, PDHA1, DLAT, and LIPT1 exhibited higher expression levels compared to normal tissues. Conversely, tumor tissues exhibited significantly reduced expression of FDX1, CAT, and EHHADH than normal (*p* < 0.05). Subgroup dot plots (Fig. 7B) showed the difference in COMMD1 expression between tumor and normal tissues. Additionally, a calibration curve highlighted a significant expression difference between the two tissue types (Fig. 7C). Finally, survival outcomes were analyzed between individuals with high and low COMMD1 expression (Fig. 7D). The implications of our findings lead to the proposition that COMMD1 holds promise as a novel gene for prognostic prediction in HCC.

### Knockdown of COMMD1 inhibits HCC cells

**Figure 8:**
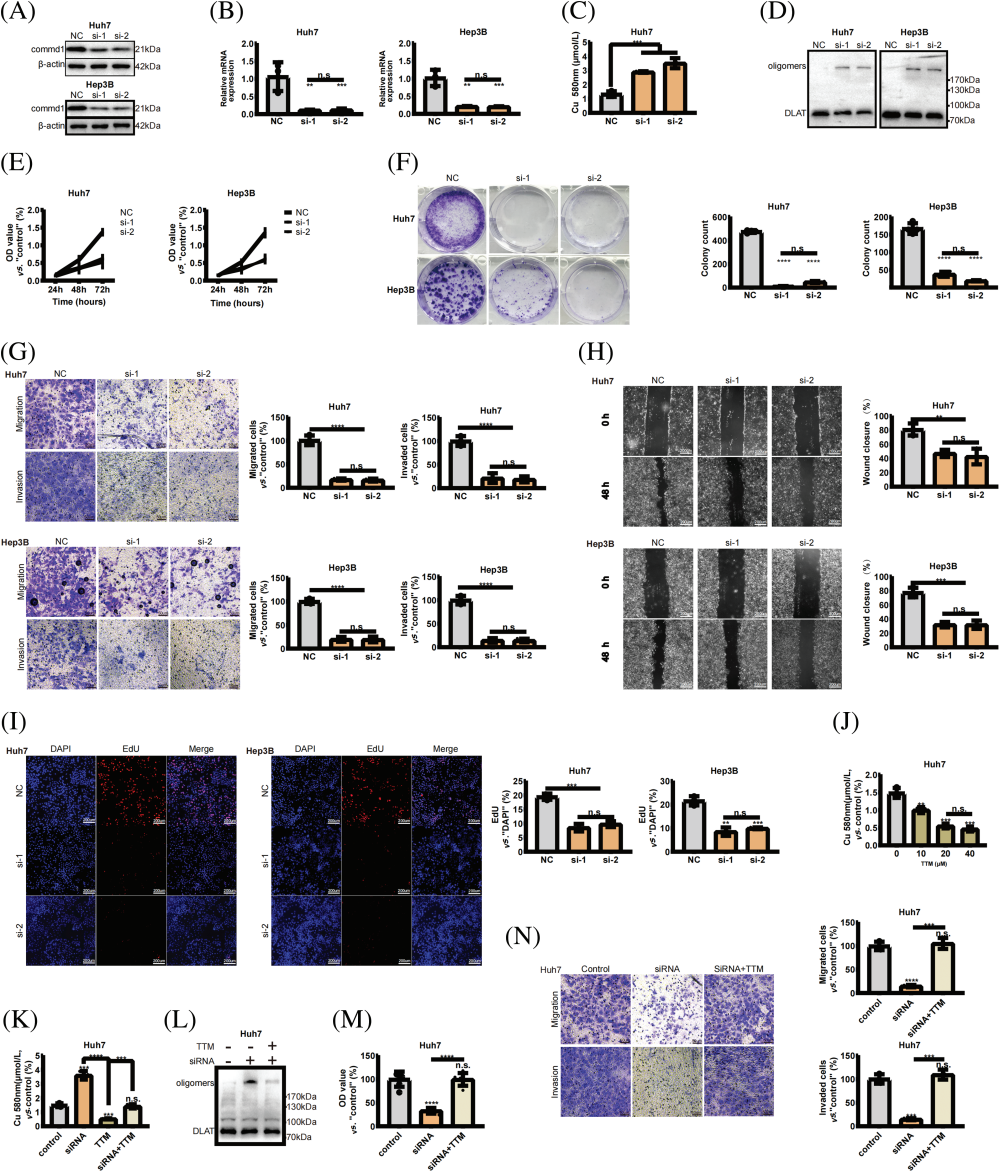
COMMD1 promotes the proliferation, invasion, and migration of HCC cells. (A and B) Western blot and qPCR were performed to assess COMMD1 knockdown efficiency in Huh7 and Hep3B cells using two different siRNAs. (C) The concentration of copper when the expression of COMMD1 decreased. (D) Western blot shows that DLAT Oligomers’ expression was significantly increased. (E) Cell proliferation of Huh7 or Hep3B cells was assessed using CCK8. (F) Cell proliferation and clonogenic capacity of control or Si-COMMD1 groups. (G) The invasion and migration ability of Huh7 and Hep3B cells. (H) Wound healing assay was conducted to compare cell migration. (I) EdU assay compared cell proliferation between control and COMMD1 knockdown cells. (J) TTM reduces intracellular copper ion concentration. (K) TTM reduces the increase in copper ion concentration caused by the knockdown of COMMD1. (L) The level of DLAT oligomers decreased with the addition of TTM. (M) Cell viability of si-COMMD1 cells was rescued by TTM. (N) The ability of migration and invasion increased when adding TTM to si-COMMD1 cells. The *p*-values were set as *p < 0.05, **p < 0.01, ***p < 0.001, and ****p < 0.0001, n.s. for not significant.

COMMD1 was knocked down in Huh7 and Hep3B cells using 2 siRNAs to determine its biological function. qPCR and WB revealed that two independent siRNA effectively silenced COMMD1 (Fig. 8A,B). The concentration of copper increased when we deleted the expression of COMMD1 (Fig. 8C). And the level of DLAT oligomers, a typical marker of cuproptosis, was significantly increased (Fig. 8D). As per the results of the CCK8 assay, in both Huh7 and Hep3B cells, depletion of COMMD1 exhibited an inhibitory effect on cancer cell proliferation (Fig. 8E). Furthermore, knockdown of COMMD1 resulted in suppression of both cell proliferation and clonogenic capacity (Fig. 8F). Concurrently, there was a significant decrease noted in the invasion and migration capabilities of both Huh7 and Hep3B cells (Fig. 8G). Subsequently, in scratch assays, the depletion of COMMD1 inhibited the rate of wound closure in Huh7 and Hep3B, indicating that COMMD1 is involved in the proliferation and migration of HCC cells (Fig. 8H). The EdU assay shows that cells’ proliferation was interrupted (Fig. 8I). At a concentration of 20 uM, TTM effectively reduces the concentration of free intracellular copper ions (Fig. 8J). TTM reduces the increase in copper ion concentration caused by the knockdown of COMMD1 (Fig. 8K) and reduces the level of DLAT oligomers (Fig. 8L). CCk8 shows that TTM can mitigate the negative effects of COMMD1 knockdown on cell viability (Fig. 8M). The addition of TTM restores the migratory and invasive capacity of COMMD1 knockdown HCC cells (Fig. 8N). These findings indicate the relevance of COMMD1 to the cuproptosis process in HCC cells, underscoring its potential as a therapeutic target for hepatocellular carcinoma.

## Discussion

The incidence of hepatocellular carcinoma shows an increasing trend every year, [18] but only 5%–15% of patients are suitable for surgical resection, leading to a poor prognosis. In order to identify supplementary biomarkers for liver cancer prognosis, we developed a prognostic model for CRGs utilizing TCGA bioinformatics (Lasso Cox regression analysis) for prognosis prediction in liver cancer. In conclusion, we have identified COMMD1 as a potential target for liver cancer therapy.

Copper, an elemental trace essential for life, plays a pivotal role in facilitating electron transfer within the oxidative respiratory chain of the tricarboxylic acid (TCA) cycle due to its robust redox properties. In 2022, Peter Tsvetkov and his team identified cuproptosis as a unique cell death pathway initiated by copper, distinct from apoptosis, necroptosis, pyroptosis, and ferroptosis [9]. Cuproptosis involves copper binding to TCA cycle components, causing proteotoxic stress and cell death [7]. Various diseases are associated with aberrant copper metabolism, including Menke’s and Wilson’s diseases, wherein recessive mutations in the ATP7A and ATP7B genes result in compromised copper excretion in healthy cells [19,20]; copper ions facilitate the accumulation of amyloid b protein in brain cells, contributing to Alzheimer’s disease [21]. Elesclomol induces cytotoxicity in diverse cancer cell types, including stem cells and drug-resistant cells. This efficacy is attributed to its impact on mitochondrial metabolism, thereby addressing therapeutic limitations associated with these tumor types [22]. Furthermore, a researcher has developed a novel nanoplatform termed GOx@[Cu(tz)], which consists of a non-porous copper(I) 1, 2, 4-triazole coordination polymer (CP). This nanoplatform is engineered with glucose oxidase (GOx) to enhance cuproptosis and photodynamic synergistic therapy [23]. The prognostic and predictive significance of CRGs in HCC patients is under investigation.

Recently, prognostic models pertaining to cuproptosis were crafted through Lasso Cox regression analysis, and their accuracy was subsequently verified. In Chen’s study, PDXK was recognized as a promising treatment target for HCC [24,25]. Using the TCGA HCC database, LASSO COX regression models were constructed with six CRGs: FDX1, CAT, CDKN2A, DLAT, LIPT1, and COMMD1. Data is split into training and testing subsets. The high-risk subset had shorter survival compared to the other. Prognosis linked to tumor stage, emphasizing treatment consideration of both tumor stage and liver function [26]. Analysis of patient’s clinical data using univariate and multifactorial Cox methods yielded results in line with existing literature, indicating that tumor stage can serve as a standalone predictor.

Genes such as FDX1, CAT, CDKN2A, DLAT, LIPT1, and COMMD1, which are associated with cuproptosis, are crucial for the growth, advancement, and spread of various cancers. Recent studies have indicated the involvement of the tumor suppressor gene p16 (CDKN2A) absence in the initial stages of tumor development [27]. According to a study published in Nature, the LIPT1 gene is crucial for the development of mammals, and its removal hinders embryonic TCA cycling, disrupts normal brain and red blood cell development, and results in embryonic death [28]. FDX1 targeted by elesclomol, is implicated in cancer cell resistance to proteasome inhibitors [29]. The investigation unveiled an inverse correlation between COMMD1 expression and CAT and DLAT while demonstrating a positive association with LIPT1 and CDKN2A expression. However, the mechanism by which these genes interact is unclear. Using Consensus matrix grouping, we divided HCC patients into two groups. A notable variation in survival duration was observed among the two cohorts.

Tumor immune evasion is crucial for tumor spread and drug resistance [30]. The tumor microenvironment forms from cancer cells interacting with normal cells, including TAMs, MDSCs, Tregs, CAFs, along with chemokines, cytokines, and signaling factors [31]. The study found that the high-risk individuals possess lower CD8T/CD4T cell levels, and higher Treg cell levels compared to the low-risk group. Cancer vaccine research focuses on boosting CTL activity to eliminate tumor cells [32]. Additionally, CD4T cells can directly kill cancer cells and act as adjuvants. CD4T cells eliminate cancer cells through the IL-6-PD1 pathway by interacting with MHC II and CTL, potentially playing a significant role in suppressing tumor growth [33,34]. Our research indicates that the reduction in CD4T and CTL cells could be linked to the onset of liver cancer. Treg cells, known for their immunosuppressive role, contribute to immune evasion in tumors by regulating immune cells (like T cells, B cells, and NK cells) through CTLA-4, IL-10, and TGF-β [35]. These results align with prior research and offer potential strategies for liver cancer treatment.

The COMMD structural domain protein family is crucial in regulating copper metabolism, NF-κB transcription factor activity, cell proliferation, and protein functionality [36]. COMMD1 was identified as an intracellular copper ion regulatory gene that maintains intracellular copper ion homeostasis by over-binding of ATB7B and [PtdIns (4,5) P2] [37]. Deletion of COMMD1 results in copper accumulation in liver cells [38]. Previous research has shown that COMMD1, the most prominent member of the COMMD family, can suppress NF-κB and HIF-mediated gene expression, reducing the aggressiveness of melanoma tumors [39]. Additionally, COMMD1 is involved in repairing DNA damage and its decreased expression hinders NSCLC cell proliferation [40]. However, the impact of COMMD1 on cancer progression has not been consistently studied, highlighting the need for further research on this protein. Our recent study identified COMMD1 as a promising prognostic marker for HCC patients, with significant upregulation observed in HCC patients compared to healthy controls, underscoring its relevance to prognosis. We propose that elevated COMMD1 expression decreases copper ion levels in hepatocellular carcinoma cells, thereby diminishing the cytotoxic effects of cuproptosis on these cells. Takamitsu Miyayama and Amila Suraweera found that COMMD1 knockdown decreased ATP7B expression and enhanced the effectiveness of cisplatin and radiotherapy on cancer cells [40,41]. This also provides a new idea for the therapy of hepatocellular carcinoma.

In the high- and low-risk patient groups, GO and KEGG methods identified distinct genes significantly enriched in functions related to DNA replication and disrupted cell cycle, contributing to cancer cell proliferation [42]. ATP7B and COMMD1 regulate cellular copper ion levels. ATP7B relocation to the trans-Golgi network is crucial for the formation of copper-laden vesicles necessary for biliary copper excretion [43]. Furthermore, COMMD1 regulates ATP7B, facilitating its relocalization to the trans-Golgi network [41], thereby promoting transporter recycling. Brancaccio et al. suggested that disruption of copper balance could harm the process of maturing Fe-S proteins in mitochondria [44]. Furthermore, Saporito-Magriñá et al. demonstrated that copper ions have the potential to cause toxicity in mitochondria, resulting in dysfunction in the liver of rats [45]. SIRT4, a mitochondrial sirtuin, plays a crucial role in maintaining mitochondrial metabolism essential for DNA damage repair and exhibits anti-tumor properties by inhibiting mitochondrial glutamine metabolism in response to cellular DNA damage [46]. This disruption of mitochondrial metabolism could potentially decrease the invasiveness of cancerous tissue. Inhibiting autophagy can hinder mitochondrial function and impact tumor growth, with the metabolic consequences influenced by the particular oncogene and tumor suppressor gene mutations and the timing of these mutations [47]. As a result, COMMD1 could potentially influence tumor tissue invasiveness through the regulation of copper ion levels and changes in mitochondrial metabolism.

High- and low-risk patient groups underwent screening with 16 drugs to assess their IC50 values, revealing statistically significant differences. Camptothecin and its derivative irinotecan, both of which are DNA topoisomerase inhibitors, are currently being utilized in the therapy of lung and colorectal cancers [48]. Camptothecin specifically targets DNA topoisomerase I, binding at the TOP1-DNA complex interface, exemplifying interfacial inhibitors capable of reversibly trapping macromolecular complexes. Consequently, they induce replication-mediated DNA double-strand breaks, exerting an effect on the IGF1R. IGF1R can affect the tumor microenvironment, and its defective expression reduces tumor growth, proliferation, and also tumor aggressiveness [49].

When we knocked down COMMD1, we found an elevation in intracellular copper ion concentrations, along with a marked elevation in the DLAT oligomer characteristic of cuproptosis. We then demonstrate that COMMD1 can influence the biological activity of tumor cells using a series of biological experiments such as CCK8, EdU, clone formation, migration, and invasion. Western blotting and quantitative PCR (qPCR) assays demonstrated the effective silencing of COMMD1 using two independent siRNAs. Furthermore, COMMD1 silencing suppressed proliferation and clonogenicity in Huh7 and Hep3B cell lines, as evidenced by EdU staining results showing a significant decrease in hepatocellular carcinoma (HCC) cell proliferation. The transwell experiment demonstrated that reducing COMMD1 expression suppressed their ability to invade. Following this, during the wound-healing test, we observed that the absence of COMMD1 slowed down the rate of wound closure in Huh7 and Hep3B cells. Tetrathiomolybdate, a kind of copper ion chelator, was added to COMMD1 knockdown HCC cells. Following the introduction of a copper chelator, there was a reduction in dlat oligomerization, and an increase in cell viability of Huh7 cells as indicated by the CCK8 assay. The findings suggest that decreased COMMD1 expression may hinder intracellular copper ion release, potentially leading to DLAT oligomer formation within cells. Accumulation of these oligomers induces proteotoxic stress, subsequently impairing the proliferative activity of HCC cells. Treatment with exogenous copper chelators reduces intracellular DLAT oligomer levels, alleviating the adverse effects of COMMD1 knockdown. Research indicates that COMMD1 holds promise as a key gene associated with cuproptosis in HCC management.

Nevertheless, our study is subject to several limitations. The dataset utilized in our study was obtained from the TCGA HCC repository, comprising slightly over 400 cases, which may not provide comprehensive persuasive evidence. Furthermore, we randomized these data 1:1 as a training and an internal validation group and used only GSE76427 as an external validation. We should have validated the model more fully against a larger HCC dataset or a self-built data cohort. Simultaneously, the precise route by which COMMD1 triggers cuproptosis remains unknown, necessitating additional experimental research to elucidate the specific role of COMMD1 in cuproptosis.

Overall, the predictive model for hepatocellular carcinoma (HCC) developed using six CRGs demonstrates strong predictive capabilities and accuracy. Additionally, it aids in risk assessment and predicting immune response and drug sensitivity in HCC patients. Conversely, our discovery of COMMD1 as a potential target gene for hepatocellular carcinoma treatment is crucial for personalized therapy in patient management.

## Supplementary Materials

Figure S1 Table of genes for "cuproptosis" and "copper metabolism".

Figure S2 Identification of the cuproptosis-related gene by Lasso cox regression analysis in HCC. **(A, B)** LASSO Cox regression with a 10-fold cross-validation for the prognostic value of the cuproptosis-associated 6 genes, including FDX1, CAT, CDKN2A, DLAT, LIPT1, COMMD1.

Figure S3 Drug sensitivity correlated with high-and low-risk patients in hepatocellular carcinoma. **(A-P)** IC 50 value of sensitive drugs in high-and low-risk patients with HCC.

## Data Availability

All study data are in this article and additional files.

## Abbreviations

COMMD1: Copper Metabolism Domain Containing 1
WB: Western blot
TTM: Tetrathiomolybdate
CCK8: Cell Counting Kit-8
FOV: Fields of View
HCC: Hepatocellular carcinoma
GEO: Gene Expression Omnibus
TCGA: The Cancer Genome Atlas
DLAT: Dihydrolipoamide S-acetyltransferase
GDC: Genomic Data Commons
GSVA: Gene Set Variation Analysis
TTM: Tetrathiomolybdate
TCA: Tricarboxylic acid cycle
ROS: Reactive Oxygen Species

## Supplementary Materials

The supplementary materials are available online at https://doi.org/10.32604/or.2024.049772.
