# Supplementary figures and images for: A novel prognostic scoring model based on cuproptosis identifies COMMD1 as a novel therapy target for liver hepatocellular carcinoma
Source: Oncol Res. 2025 Feb 28;33(3):617–30. doi: 10.32604/or.2024.049772 (PMC11915051; doi:10.32604/or.2024.049772)

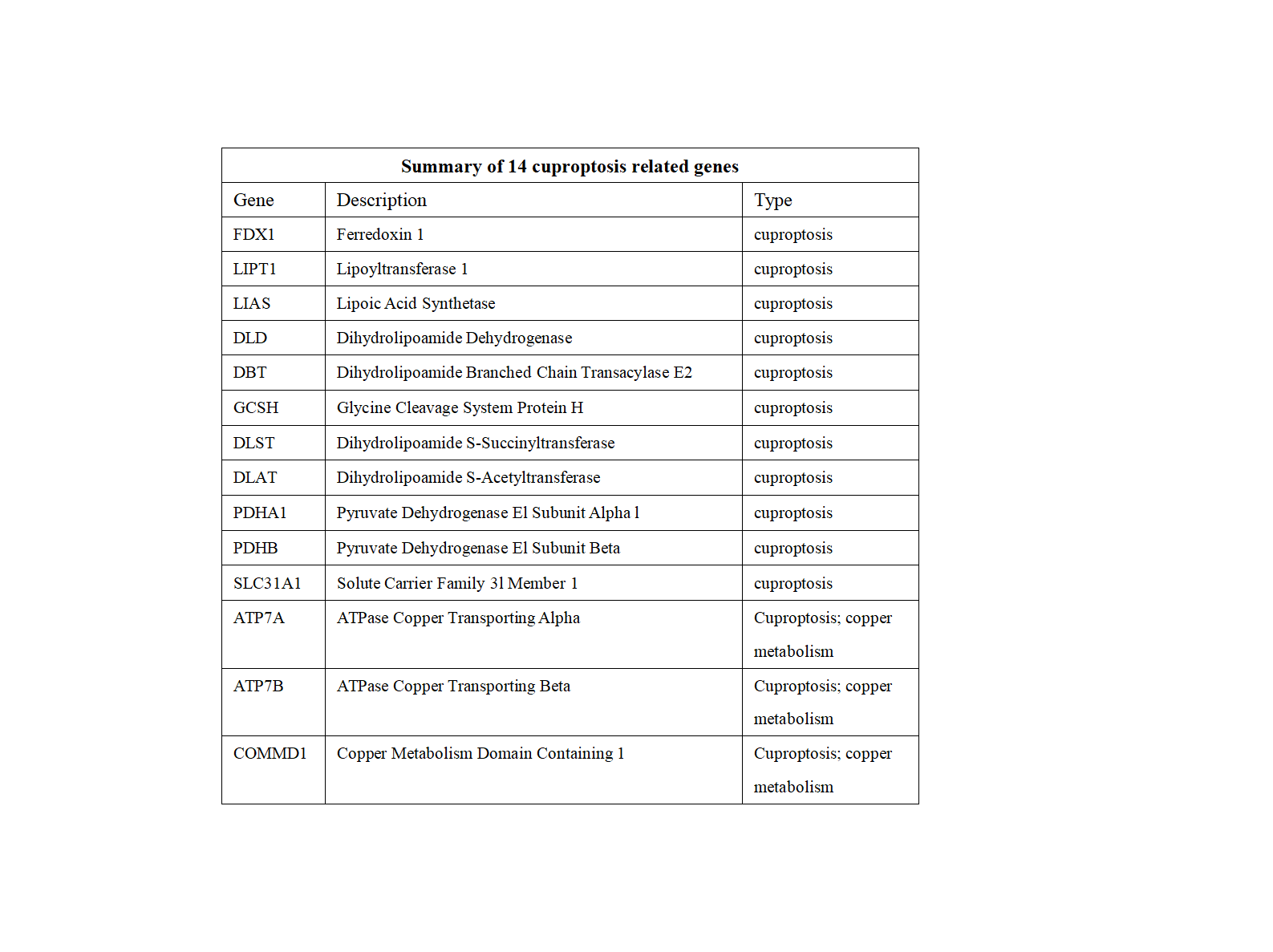

Supplement: Figure S1 [file OncolRes-33-49772-s001.tif]

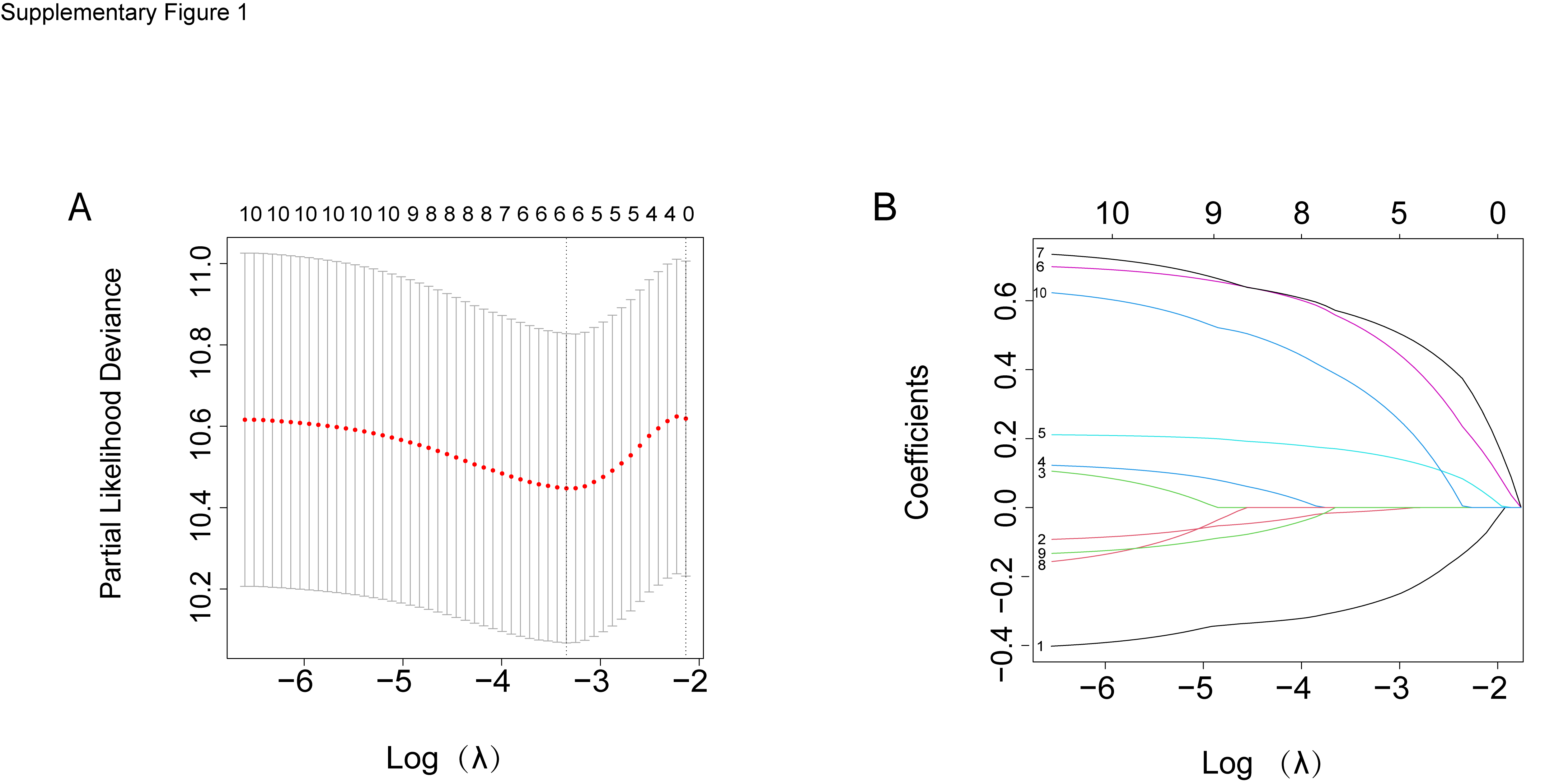

Supplement: Figure S2 — (A, B) LASSO Cox regression with a 10-fold cross-validation for the prognostic value of the cuproptosis-associated 6 genes, including FDX1, CAT, CDKN2A, DLAT, LIPT1, COMMD1. [file OncolRes-33-49772-s002.tif]
